# New Perspectives on Diagnostics, Prognosis, and Therapy in Aggressive Endocrine Tumours

**DOI:** 10.1155/2012/820938

**Published:** 2012-12-05

**Authors:** Marialuisa Appetecchia, Bruce H. R. Wolffenbuttel, Saadi Al Jadir

**Affiliations:** ^1^Endocrinology Unit, Regina Elena National Cancer Institute, 53 Elio Chianesi Street, 00144 Rome, Italy; ^2^Department of Endocrinology, University Medical Center Groningen, Groningen, The Netherlands; ^3^Endocrinology & Diabetes Unit, Department of Internal Medicine, Fujairah Hospital, Fujairah, UAE

The research process is not entirely complete and sounds until the results are validated and transmitted to an appropriate target audience through modern publication.

Only through well-designed publication of results in established peer-reviewed journals or scientific congresses can new ideas and research findings be disseminated and effectively incorporated into clinical practice and eventually improvement in physician's performance and patient's health outcome.

We had ensured that all published reports of research have been reviewed by suitably qualified reviewers (e.g., including statistical review where appropriately needed).

Therefore, we are intended in our journal to achieve successful medical writing in order to serve as the optimal pathway to advance medical knowledge, promote critical thinking, incite scientific debate by involving a lot of expertise in our publications, and maintain the high degree of scientific and professional quality besides considering the continuing progress in different fields, modern medicine. 

Endocrine tumors are a mixed group of diseases in which neoplastic cells are found in tissues of the endocrine system, which includes the thyroid, adrenal, pancreas, parathyroid, and pituitary glands.

 Endocrine-related neoplasm has been enlarged to the domain of endocrine system to neural tissue as the embryonic development is closely interrelated; therefore, neuroendocrine tumors will be literally included in our researches, neuroendocrine tumors (Merkel cell, islet cell, MCT, pheochromocytoma, carcinoids etc.).

Most of these tumors appear as sporadic on one hand, and on other they might be clusters or familial part of genetic syndrome like MEN1, MEN2, or other more rare disorders.

These tumors ordinarily secrete hormones of the glands that have arisen from, and sometimes they are nonsecretors, majority are benign and others are malignant; some of these neoplasms tend to have indolent course, others are short and aggressive, as their clinical presentation and management vary accordingly. It is not surprising to find these tumors in unusual or ectopic sites that eventually rendered their topography and presentation difficult.

Last decades had shown a great development and tremendous successes in eruption of variety of biochemical; immunochemical; imaging techniques and most of these tumors showed their biological activity before their size could be detected by the conventional diagnostic testing. Currently, most of endocrine neoplasms can be successfully localized and subsequently have made their managements feasible. 

In this special issue, we had tried to incorporate many aspects in studying these tumors including genetics behaviors, diagnosis, clinical course, new modalities in diagnosis, management, and surgical approaches as well as chemotherapy and specific palliative procedures in metastatic disease.

Clinical studies and review articles in this special issue have demonstrated the most recent updates in diagnosis and new treatment strategies, besides the enormous improvement in molecular biology of some relatively uncommon tumors that certainly will be of utmost importance to the practicing practitioners.

Thyroid cancer is the most common endocrine tumor. Besides standard treatment for differentiated thyroid cancer, the research for new therapies in advanced nonmedullary and medullary thyroid carcinomas, with the advent of tyrosine kinase inhibitors as well as antiangiogenic inhibitors, those patients could have an advantage with new target therapy. 

In adrenocortical carcinoma radical surgery is considered the therapy of choice in the first stages of ACC. Mitotane, an adrenolytic drug with significant toxicity and unpredictable therapeutic response, is used in the treatment of ACC. Although, treatment for this aggressive cancer is still ineffective. Over the past years, the growing interest in ACC has contributed to the development of therapeutic strategies in order to contrast the neoplastic spread.

The efficacy of radiolabeled somatostatin analogues in patients with advanced neuroendocrine tumors had been illustrated in one article and clearly had exhibited that overall tumor response rate was appreciable, and PRRT is a promising perspective for patients with advanced NETs.

Cushing's syndrome whenever surgery is not curative, management of patients requires a major effort to control excess cortisol and associated symptoms. A multidisciplinary approach should be adopted. For aggressive ACTH dependent, several drugs are able to reduce cortisol levels. Their mechanism of action involves blocking adrenal steroidogenesis and novel chemotherapeutic agents (temozolomide and tyrosine kinase inhibitors) which have a significant activity against aggressive pituitary or ectopic tumors.

Recent genetic studies of malignant pheochromocytomas/paragangliomas have highlighted the main pathways involved in pathogenesis, thus suggesting the use of targeted therapy which, nevertheless, still has to be validated. Large collaborative studies on tissue specimens and clinical trials in large cohorts of patients are necessary to achieve better therapeutic tools and improve patient prognosis.

Review article about insulinoma had highlighted many aspects on this small neoplasm, including biological activity, new emerging imaging techniques in localizing the tumor especially in resectable ones, molecular pathogenesis of malignant disease, and treatment modalities for aggressive course, and put consequent hypoglycemia under control.

Merkel cell carcinoma is a rare and aggressive neuroendocrine tumor of the skin. Wide surgical excision must be associated with radiotherapy in early stages. In advanced disease, chemotherapy is the standard option despite the short duration of responses and poor quality of life. 

Multiple endocrine neoplasias (MENs) are clinical inherited syndromes affecting different endocrine glands. Three different patterns of MEN syndromes can occur (MEN 1, MEN 2A, and MEN 2B). MEN 1 is characterized by the neoplastic transformation of the parathyroid glands, pancreatic islets, anterior pituitary, and gastrointestinal tract. Therapeutic approaches are different according to the different endocrinopathies. In MEN 2 syndromes, the medullary thyroid cancer is almost invariably present and can be associated with pheochromocytoma and/or multiple adenomatosis of parathyroid glands with hyperparathyroidism. Although surgery is the main option, nevertheless, 30% of MTC patients, especially in MENs 2B and 2A, are not cured by surgery. Recently, developed molecular therapeutics that target the RET pathway have shown very promising activity in clinical trials of patients with advanced MTC. 

There is growing evidence of the role of IGF system dysregulation in endocrine neoplasms, and we will discuss the possible implications of these findings for tumor prevention and treatment, with a major focus on cancers from the thyroid, adrenal, and ovary, which are the most extensively studied. However, multiple molecular abnormalities of the IGF system frequently occur in endocrine neoplasms and may have a role in tumorigenesis as well as in tumor progression and resistance to therapies. 

*Marialuisa Appetecchia* *Marialuisa Appetecchia* *Bruce H. R. Wolffenbuttel* *Bruce H. R. Wolffenbuttel* *Saadi Al Jadir* *Saadi Al Jadir*

